# Pioglitazone is equally effective for diabetes prevention in older versus younger adults with impaired glucose tolerance

**DOI:** 10.1007/s11357-016-9946-6

**Published:** 2016-09-01

**Authors:** Sara E. Espinoza, Chen-pin Wang, Devjit Tripathy, Stephen C. Clement, Dawn C. Schwenke, Mary Ann Banerji, George A. Bray, Thomas A. Buchanan, Robert R. Henry, Abbas E. Kitabchi, Sunder Mudaliar, Frankie B. Stentz, Peter D. Reaven, Ralph A. DeFronzo, Nicolas Musi

**Affiliations:** 1 Barshop Institute for Longevity and Aging Studies, University of Texas Health Science Center at San Antonio, 7703 Floyd Curl Drive, San Antonio, TX 78223 USA; 2 Geriatrics Research, Education and Clinical Center, South Texas Veterans Health Care System, 7400 Merton Minter Blvd., San Antonio, TX 78229 USA; 3 Texas Diabetes Institute, University of Texas Health Science Center at San Antonio, 7703 Floyd Curl Dr, San Antonio, TX 78229 USA; 4 South Texas Veterans Health Care System, 7400 Merton Minter Blvd., San Antonio, TX 78229 USA; 5 Department of Medicine Division of Endocrinology and Metabolism, Georgetown University, 3700 O St NW, Washington, DC, 20057 USA; 6 Arizona State University, Tempe, AZ 85281 USA; 7 Department of Medicine Division of Endocrinology, State University of New York Downstate Medical Center, 450 Clarkson Ave, Brooklyn, NY 11203 USA; 8 Pennington Biomedical Research Center, Louisiana State University, Baton Rouge, LA 70803 USA; 9 Department of Medicine Division of Endocrinology and Diabetes, University of Southern California, Los Angeles, CA USA; 10 Department of Medicine Division of Endocrinology and Metabolism, University of California San Diego, 9500 Gilman Dr, La Jolla, CA 92093 USA; 11 Department of Medicine Division of Endocrinology, Diabetes and Metabolism, University of Tennessee Health Science Center, 920 Court Ave, Memphis, TN 38163 USA; 12 Phoenix Veterans Affairs Health Care System, 650 E Indian School Rd, Phoenix, AZ 85012 USA

**Keywords:** Geriatrics, Diabetes, Prevention, Endocrinology

## Abstract

To determine the efficacy of pioglitazone to prevent type 2 diabetes in older compared to younger adults with pre-diabetes. Six hundred two participants with impaired glucose tolerance (IGT) were randomized in double blind fashion to placebo or pioglitazone for diabetes prevention in the ACT NOW study (NEJM 364:1104–1115, 2011). Cox proportional hazard regression was used to compare time to development of diabetes over a mean of 2 years between older (≥61 years) and younger participants. We compared effects of pioglitazone versus placebo on metabolic profiles, inflammatory markers, adipokines, β cell function (disposition index), insulin sensitivity (Matsuda index), and body composition by ANOVA. Diabetes incidence was reduced by 85 % in older and 69 % in younger subjects (*p* = 0.41). β cell function (disposition index) increased by 35.0 % in the older and 26.7 % in younger subjects (*p* = 0.83). Insulin sensitivity (Matsuda index) increased by 3.07 (5.2-fold) in older and by 2.54 (3.8-fold) in younger participants (*p* = 0.58). Pioglitazone more effectively increased adiponectin in older versus younger subjects (22.9 ± 3.2 μg/mL [2.7-fold] vs. 12.7 ± 1.4 μg/mL [2.2-fold], respectively; *p* = 0.04). Younger subjects tended to have a greater increase in whole body fat mass compared to older subjects (3.6 vs. 3.1 kg; *p* = 0.061). Younger and older subjects had similar decreases in bone mineral density (0.018 ± 0.0071 vs. 0.0138 ± 0.021 g/cm^2^). Younger and older pre-diabetic adults taking pioglitazone had similar reductions in conversion to diabetes and older adults had similar or greater improvements in metabolic risk factors, demonstrating that pioglitazone is useful in preventing diabetes in older adults.

**Electronic supplementary material:**

The online version of this article (doi:10.1007/s11357-016-9946-6) contains supplementary material, which is available to authorized users.

## Background

The prevalence of diabetes increases dramatically with age and it is estimated that 25–30 % of individuals over the age of 65 have type 2 diabetes (Centers for Disease Control and Prevention 2014). Impaired glucose tolerance (IGT) also is highly prevalent in older adults, affecting ∼35 % of individuals >65 years (Cowie et al. 2009). Further, individuals with IGT are at high risk of converting to diabetes (5–10 % per year) (Tabak et al. 2012) and manifest similar pathophysiologic disturbances to individuals with type 2 diabetes (DeFronzo 2009). Diabetes is a major contributor to adverse health outcomes with aging (Blaum et al. 2003) and is a significant predictor of disability (Gregg et al. 2000) and frailty (Espinoza et al. 2012), both of which pose significant public health burdens. Therefore, interventions to prevent or delay diabetes in older adults are important in improving aging outcomes as the numbers of older adults who either are living with diabetes or are at risk of developing diabetes increases (Sloan et al. 2008).

Lifestyle modification and some anti-diabetic agents (metformin, acarbose, liraglutide) have been useful in preventing diabetes in prediabetic adults at high risk of developing diabetes (Chiasson et al. 2002; DeFronzo et al. 2011; Diabetes Prevention Program Research Group 2002; Pi-Sunyer et al. 2015). Thiazolidinediones also delayed diabetes progression among subjects with IGT in three placebo-controlled multicenter trials (Buchanan et al. 2002; DeFronzo et al. 2011; DREAM Trial Investigators et al. 2006). In the ACT NOW Study, pioglitazone reduced the conversion of IGT to diabetes by 72 % (DeFronzo et al. 2011).

To design and apply strategies to prevent type 2 diabetes in older adults, it is necessary to determine whether interventions that delay diabetes in the general population are equally effective as people age. A secondary analysis of the Diabetes Prevention Program (DPP) showed that older adults (ages 60 to 85 years) were more responsive to intensive lifestyle intervention than to metformin, compared to younger adults (ages 25 to 59) (Diabetes Prevention Program Research Group 2006), as determined by oral glucose tolerance testing. These findings were thought to be, in part, related to the greater weight loss and more active participation in lifestyle modification activities among older participants and possibly related to differences in the pathophysiology of insulin resistance in aging individuals (Basu et al. 2003).

The goal of the present study was to evaluate whether aging modifies the effectiveness of pioglitazone for the prevention of diabetes. For this purpose, we compared responses between older (ages 61 through 86 years) and younger (ages 18 through 60 years) subjects in the ACT NOW trial.

## Methods

### Subjects

Subjects were 602 participants from the ACT NOW trial aged 18 or older. We selected the cut point of ages 61 through 86 years for the older group and ages 18 through 60 years for the younger group because age 61 corresponds to the top 25th percentile for age distribution in this study sample. Patients were eligible for the ACT NOW study if they had a fasting plasma glucose concentration between 95 and 125 mg/dl (5.3 and 6.9 mmol/l) and at least one other risk factor for diabetes (DeFronzo et al. 2009). All subjects had IGT, defined as a 2-h plasma glucose between 140 and 199 mg/dl [7.8 to 11.0 mmol/l] during a single oral glucose tolerance test (OGTT) (American Diabetes Association 2008) as published previously (DeFronzo et al. 2009, 2011). The first participant was recruited in January 2004, and enrollment was completed in March 2006. Participants were followed until they reached the primary end point of diabetes, withdrew from the study, were lost to follow-up, or completed the study.

### Study design

The study design and protocol have been described previously (DeFronzo et al. 2009). Briefly, eight centers participated in this investigator-initiated study, which was approved by the Institutional Review Board at each site. Written informed consent was obtained from all participants. All results were transmitted to the Data Coordinating Center in Phoenix, Arizona, where they were recorded and audited and then sent to the Data Analysis Center in San Antonio. Takeda Pharmaceuticals provided financial support for the study but had no access to the data. After eligibility for the study was ascertained, participants underwent randomization according to center and sex. Subjects received 30 min of dietary instruction consistent with the goals of the DPP (Diabetes Prevention Program Research Group 2002).

After enrollment, participants were asked to fast overnight and a 75-g OGTT was performed at ∼8 a.m. the next day. Plasma samples were collected every 15 min for 2 h for measurement of plasma glucose and insulin concentrations. Additional baseline assessments included measurements of blood pressure, height, weight, waist circumference, hemoglobin A1c (HbA1c), and lipids. Dual-energy X-ray absorptiometry (DEXA) scans were performed using a Hologic QDR 4500A instrument (Watertown, MA, USA) as previously described (Bray et al. 2013). Five of the eight study centers participated in the DEXA substudy, which had 232 participants.

Following completion of the OGTT, participants received pioglitazone 30 mg/day or a placebo. After 1 month, the dose of pioglitazone was increased to 45 mg/day as tolerated. Participants returned at 2, 4, 6, 8, 10, and 12 months during the first year of the study and every 3 months thereafter. At each visit, weight, blood pressure, and pulse were measured and the extent of edema was graded as described (DeFronzo et al. 2011). Fasting plasma glucose was measured at each follow-up visit. HbA1c and plasma lipids were measured every 6 months, and the OGTT was repeated annually. Plasma C-reactive protein (CRP), monocyte chemotactic protein (MCP)-1, plasminogen-activating inhibitor (PAI)-1, tumor necrosis factor (TNF)-α, interleukin (IL)-6, leptin, and adiponectin were measured at baseline and at study end.

### Conversion of impaired glucose tolerance to diabetes

The primary outcome was the development of diabetes (defined as a fasting plasma glucose level ≥ 126 mg/dl [≥7.0 mmol/l] or a 2-h glucose level ≥ 200 mg/dl [11.1 mmol/l]); a repeat OGTT was performed to confirm the diagnosis. If the diagnosis was not confirmed, participants continued their assigned therapy.

### Calculations

Insulin sensitivity (Matsuda index) was derived from plasma glucose and insulin measurements obtained during the OGTT as previously described (Matsuda and DeFronzo 1999). β cell function was determined with the insulin secretion/insulin resistance (disposition) index calculated as (Δ^I^0–120/Δ^G^0–120) × (Matsuda index) during the OGTT, where Δ^I^0–120/Δ^G^0–120 represents the increment in plasma insulin and glucose concentrations during the 120-min OGTT (Abdul-Ghani et al. 2006). Plasma glucose, insulin, lipids, HbA1c, CRP, MCP-1, PAI-1, TNF-α, IL-6, leptin, and adiponectin were measured as previously described (DeFronzo et al. 2013, 2009).

### Statistical analysis

Baseline characteristics were examined by age group (stratified according to age: ≥ 61 years vs. <61) using chi-squared tests for categorical variables and *t* tests for continuous variables. Linear regression analysis was used to examine study outcomes in response to pioglitazone versus placebo at study end by age group. Outcomes assessed were change in insulin sensitivity (Matsuda index), fasting plasma glucose, fasting insulin, lipids, HbA1c, adipokines, and markers of inflammation. Additional regression analyses were performed with covariate adjustment for sex and baseline value of each individual outcome (i.e., change in adiponectin was adjusted for sex and baseline adiponectin value). Log transformation was used for non-normally distributed outcome variables. Cox proportional hazard models were used to examine time to diabetes conversion by age category. These analyses were adjusted for sex and baseline measures.

## Results

### Subject characteristics

Baseline data stratified by age and treatment assignment are shown in Table 1. There were no significant differences between groups in either the young or older participants for any characteristics. Overall, regardless of treatment assignment, older participants were more likely to be female (56 vs. 36 %, *p* < 0.001), but did not differ by race or ethnic group compared to younger participants. At baseline, older participants had lower BMI (32.5 ± 5.0 vs. 35.0 ± 6.5 kg/m^2^, *p* < 0.0001), higher systolic blood pressure (134 ± 16 vs. 126 ± 16 mmHg, *p* < 0.0001), lower fasting insulin (8.9 ± 8.3 vs. 11.4 ± 9.1 μ U/mL, *p* < 0.001), lower LDL cholesterol (99 ± 28 vs. 108 ± 31 mg/dL, *p* = 0.001), higher HDL cholesterol (42 ± 11 vs. 40 ± 10 mg/dl, *p* = 0.007), and lower total cholesterol (166 ± 33 vs. 173 ± 35 mg/dL, *p* = 0.028) compared to younger participants. Overall, compared to younger participants, older participants had slightly higher insulin sensitivity as measured by the Matsuda index at baseline (4.4 ± 2.7 vs. 3.8 ± 2.7, *p* = 0.0008).

**Table 1:** Characteristics by treatment and age groups at study baseline

	Young age < 61			Older age ≥ 61				
	Pioglitazone *N* = 214	Placebo *N* = 220	*p* value	Pioglitazone *N* = 88	Placebo *N* = 80	*p* value	Total *N* = 602	*p* value age difference (young vs. old)^a^
	*N* (%) or mean (SD)	*N* (%) or mean (SD)		*N* (%) or mean (SD)	*N* (%) or mean (SD)		*N* (%) or mean (SD)	
Age	47.4 (8.6)	46.2 (9.2)	0.083	65.9 (4.9)	66.3 (4.9)	0.637	52.3 (11.8)	<0.0001
Race			0.240			0.809		
White	161 (75.2)	178 (80.9)		72 (81.8)	67 (83.8)		478 (79.4)	0.8094
Black	44 (20.6)	34 (15.5)		13 (14.8)	11 (13.8)		102 (16.9)	
Asian	5 (2.3)	8 (3.6)		2 (2.3)	2 (2.5)		17 (2.8)	
Pacific Islander	1 (0.5)	0 (0.0)		0 (0.0)	0 (0.0)		1 (0.2)	
Native American	2 (0.9)	0 (0.0)		1 (1.1)	0 (0.0)		3 (0.5)	
Other	1 (0.5)	0 (0.0)		0 (0.0)	0 (0.0)		1 (0.2)	
Sex, female	80 (37.4)	78 (35.5)	0.676	47 (53.4)	47 (58.8)	0.486	252 (41.9)	<0.001
Pioglitazone group							302 (50.2)	0.5583
BMI (kg/m^2^)	34.6 (6.4)	32.3 (6.7)	0.333	32.5 (5.1)	32.5 (5.0)	0.996	34.3 (6.2)	<0.0001
Body surface area (m^2^)	2.1 (0.3)	2.1 (0.3)	0.317	2.1 (0.2)	2.1 (0.2)	0.471	2.10 (0.25)	0.0710
Waist circumference (cm)	106.3 (14.1)	107.7 (15.1)	0.347	106.0 (11.4)	105.9 (13.4)	0.957	106.7 (14.0)	0.4220
Systolic blood pressure (mmHg)	125.7 (16.3)	125.5 (15.8)	0.893	131.5 (15.5)	136.4 (16.3)	0.047	128 (16)	<0.0001
Diastolic blood pressure (mmHg)	74.5 (9.9)	74.0 (10.4)	0.578	72.4 (8.1)	74.2 (10.2)	0.220	74 (10)	0.3395
Fasting plasma glucose (mg/dL)	104.7 (7.9)	104.7 (7.9)	0.961	104.9 (7.0)	104.0 (7.1)	0.389	105 (8)	0.7677
Fasting insulin (mU/mL)	11.1 (8.2)	11.6 (9.9)	0.565	9.1 (9.1)	8.8 (7.5)	0.835	10.7 (8.9)	<0.001
Hemoglobin A1c (%)	5.5 (0.4)	5.5 (0.4)	0.741	5.6 (0.5)	5.5 (0.4)	0.130	5.5 (0.4)	0.126
Hemoglobin A1c (mmol/mol)	36	36		38	36		36	
Triglycerides (mg/dL)	125.8 (63.5)	119.1 (56.0)	0.253	118.0 (47.3)	123.9 (65.4)	0.506	122 (59)	0.9569
Total cholesterol (mg/dL)	171.0 (34.6)	173.9 (35.5)	0.400	162.6 (32.4)	168.8 (33.6)	0.229	171 (35)	0.0282
HDL cholesterol (mg/dL)	39.7 (10.3)	39.7 (10.1)	0.978	41.3 (11.6)	43.5 (10.9)	0.210	40 (11)	0.0073
LDL cholesterol (mg/dL)	106.2 (30.7)	110.3 (31.8)	0.178	97.7 (29.2)	101.1 (28.0)	0.454	105.8 (30.8)	0.0012
Matsuda index	3.7 (2.6)	3.8 (2.6)	0.894	4.4 (2.5)	4.4 (3.0)	0.914	3.9 (2.6)	0.0008
Disposition index	3.2 (1.9)	3.2 (1.9)	0.933	3.4 (1.6)	3.4 (1.5)	0.865	3.23 (1.79)	0.1206
Glucose area under the curve during 120 min OGTT	1.1 (0.6)	1.1 (0.7)	0.913	1.0 (0.6)	1.0 (0.6)	0.440	1.05 (0.6)	0.1599
IL-6 (pg/mL)	7.2 (50.4)	1.7 (1.6)	0.188	7.2 (24.9)	2.1 (1.4)	0.121	4.5 (31.3)	0.0304
Leptin (pg/mL)	35.6 (23.1)	36.7 (25.7)	0.719	34.2 (26.3)	34.8 (27.9)	0.908	35.7 (25.2)	0.086
MCP-1 (pg/mL)	136.9 (56.6)	123.7 (51.4)	0.038	156.4 (71.4)	159.2 (55.8)	0.811	138 (59)	<0.0001
TNF-α (pg/mL)	4.2 (3.7)	3.7 (1.6)	0.152	4.9 (1.9)	4.7 (1.6)	0.506	4.2 (2.6)	<0.0001
Adiponectin (μg/mL)	11.0 (6.7)	11.1 (7.8)	0.860	12.6 (7.0)	13.6 (8.2)	0.454	11.7 (7.4)	0.0011
PAI-1 (ng/mL)	15.1 (8.8)	15.9 (8.3)	0.400	15.1 (12.2)	13.6 (5.9)	0.404	15.2 (8.9)	0.288
CRP (mg/L)	3.4 (3.9)	3.1 (3.8)	0.695	4.9 (4.9)	2.7 (2.9)	0.264	3.4 (3.9)	0.9667

^a^ *p* value for age difference between young and older participants across treatment groups

There were no significant differences in plasma inflammatory biomarkers between pioglitazone and placebo groups in the young versus older age groups. However, older participants had higher plasma IL-6 (4.7 ± 18.0 vs. 4.4 ± 35.4 pg/mL, *p* = 0.030), MCP-1 (158 ± 64 vs. 130 ± 54 pg/mL, *p* < 0.0001), TNF-α (4.8 ± 1.8 vs. 3.9 ± 2.8 pg/mL, *p* < 0.0001), and adiponectin (13.0 ± 7.6 vs. 11.1 ± 7.2 μg/mL, *p* = 0.0011) concentration at baseline. There were no overall age differences in circulating levels of leptin, PAI-1, or CRP compared with younger participants.

Baseline body composition and bone density data for participants in the DEXA substudy are shown in Supplemental Table 1. Older subjects had higher whole body fat percentage (41.9 ± 6.2 vs. 38.8 ± 7.8 %, *p* = 0.023) and lower bone mineral density (0.95 ± 0.09 vs. 1.02 ± 0.11 g/cm^2^, *p* = 0.0025) compared to younger participants.

### Effect of treatment on conversion to diabetes

**Fig. 1:**
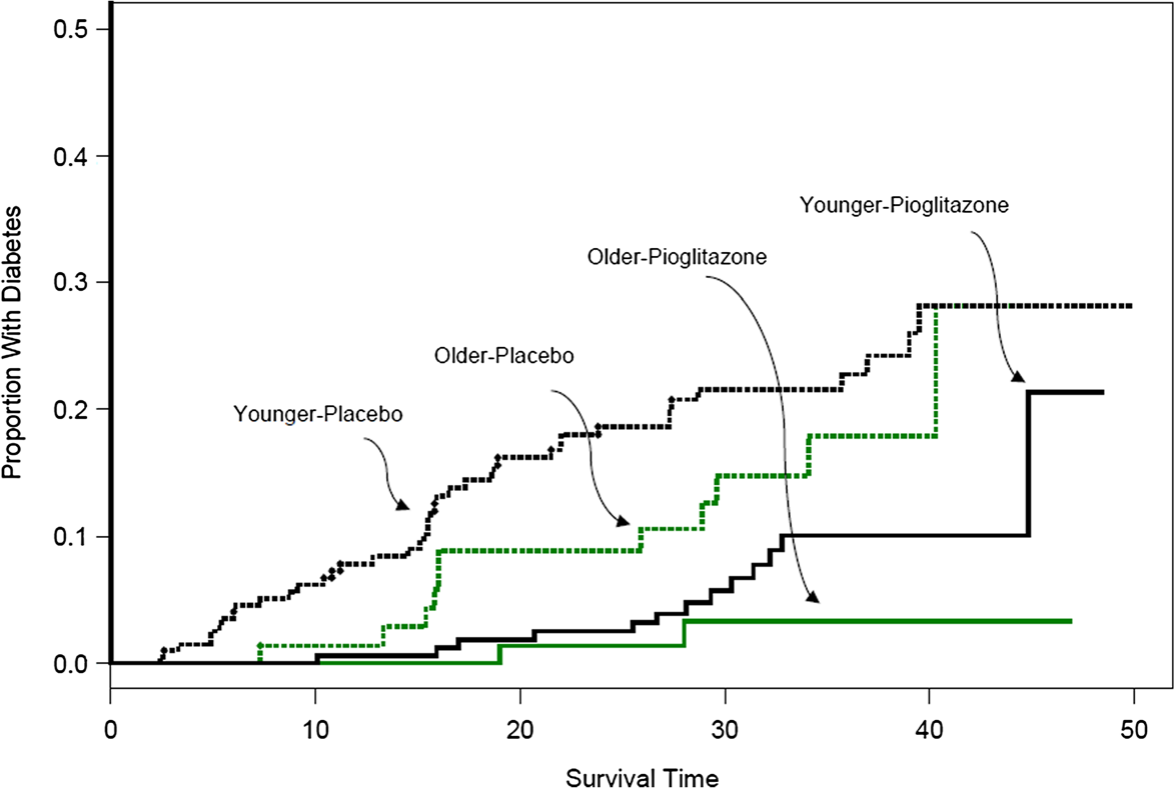
Kaplan-Meier curve for time to conversion to diabetes by treatment group (pioglitazone vs. placebo) and age group (age ≥ 61 vs. <61 years)

As previously reported, pioglitazone reduced the incidence of diabetes by 72 % overall (HR = 0.28, 95 % CI 0.16–0.49; *p* < 0.001) (10). After adjustment for age and sex, this finding did not change significantly (HR = 0.29, 95 % CI 0.16–0.49; *p* < 0.01). In older participants, diabetes incidence was reduced by 84 % (HR = 0.16, 95% CI 0.05–0.50; *p* < 0.01); and in younger subjects diabetes incidence was reduced by 69 % (HR = 0.31, 95% CI 0.17–0.57, *p* < 0.01) (Fig. 1). Diabetes incidence did not differ by age (*p* = 0.41).

### Effect of pioglitazone on laboratory and physiologic parameters

**Fig. 2:**
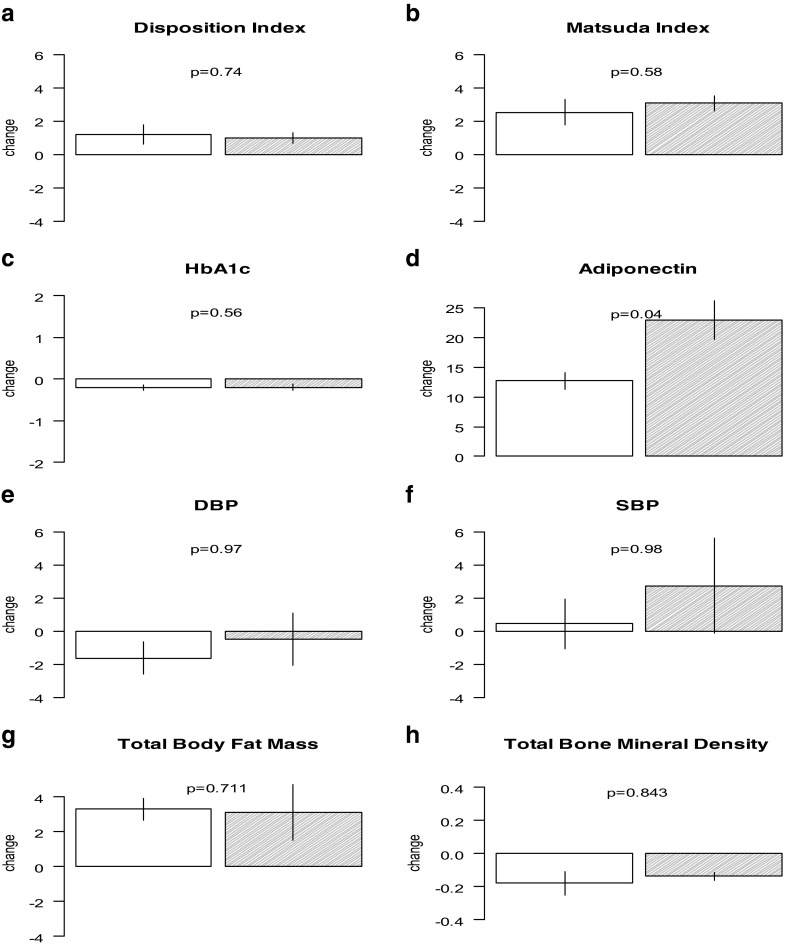
Change with pioglitazone compared to placebo in: **a** disposition index, **b** Matsuda index, **c** hemoglobin A1c (HBA1c), **d** adiponectin, **e** diastolic blood pressure(DBP), **f** systolic blood preassure (SBP), **g** total body fat mass, **h** total bone mineral density by age group. The *open boxes* indicate the younger age group (age < 61 years) and the *filled boxes* represent the older age group (age ≥ 61 years)

As previously reported, pioglitazone reduced fasting plasma glucose, insulin, hemoglobin A1c, triglycerides, PAI-1, IL-6, TNF-α and increased insulin sensitivity, plasma high density lipoprotein (HDL), and adiponectin in the overall cohort (DeFronzo et al. 2013, 2011; Saremi et al. 2013). For all indices in the unadjusted analyses, older and younger adults responded similarly to pioglitazone (Fig. 2 and Supplemental Table 3). However, in analyses adjusted for sex and baseline value, adiponectin was increased more in older compared to younger subjects taking pioglitazone (22.94 ± 3.19 μg/mL [2.72-fold increase] vs. 12.70 ± 1.43 μg/mL [2.23-fold increase], *p* = 0.04). In pioglitazone-treated subjects, β cell function (disposition index) increased by 0.98 (1.2-fold) in older adults (*p* = 0.08) and by 1.2 (1.19-fold) in younger subjects (*p* < 0.01). There was no difference in response by age group (*p* = 0.74). Pioglitazone improved insulin sensitivity (Matsuda index) in the overall cohort (DeFronzo et al. 2011). The Matsuda index of insulin sensitivity increased to 3.07 (5.2-fold increase) in older adults taking pioglitazone versus placebo (*p* < 0.01) and to 2.54 (3.8-fold increase) in younger subjects (*p* < 0.01); however, there was no significant difference in increased insulin sensitivity in older versus younger subjects taking pioglitazone (*p* = 0.58).

### Body composition

Pioglitazone increased whole body fat mass in the overall ACT NOW cohort (Bray et al. 2013), and the increment from baseline in body fat was similar in younger (by 3.62 ± 0.63 kg [2.6-fold], *p* < 0.001) and older (by 3.10 ± 1.6 kg [2.6-fold], *p* = 0.061) subjects (*p* = 0.751 between groups) (Supplemental Table 3). Pioglitazone reduced total body bone mineral density in the overall cohort (Bray et al. 2013). The decrease in total bone mineral density with pioglitazone was similar in younger (by 0.018 ± 0.0071 g/cm^2^, *p* = 0.013) and older (by 0.0138 ± 0.021 g/cm^2^, *p* = 0.521) subjects. However, this effect did not differ by age group (*p* = 0.835 between groups).

### Adverse events

Adverse events were low in both groups (Supplemental Table 4). No differences between groups were seen with respect to changes in peripheral edema, hematuria, body mass index, waist circumference, or fractures or other adverse events. There were also no age group differences in the incidence of adverse events.

## Discussion

Older adults are at increased risk of developing diabetes, and those with IGT are at the highest risk. In this study, we examined whether the efficacy of pioglitazone for diabetes prevention and metabolic responses differed by age. Overall, older and younger participants had similar responses for most outcomes. Notably, pioglitazone was highly effective in reducing conversion to diabetes in older subjects, similar to responses in younger individuals.

Age-dependent declines in both β cell function and insulin sensitivity play major roles in the deterioration of glucose homeostasis that occurs with advancing age (Chang et al. 2006; Ghosh et al. 2011). Pioglitazone is thought to improve β cell function and ameliorate insulin resistance through its action on the peroxisome proliferator-activated receptor (PPAR)-γ (DeFronzo et al. 2013). We previously showed that the risk of developing diabetes in the ACT NOW Study was closely related to waning β cell function as determined by the insulin secretion/insulin resistance (IS/IR) index and that an improved IS/IR index with pioglitazone was the strongest predictor for reduced risk of conversion to diabetes. In the present study, the IS/IR index improved similarly in older and younger adults. Thus, while β cell function seems to decline progressively with advancing age (Chang et al. 2006), β cells from older participants still responded appropriately to the PPAR-γ agonist. Similarly, in older and younger subjects, pioglitazone was equally effective in improving insulin sensitivity, a major factor contributing to diabetes prevention (DeFronzo et al. 2009, 2011).

While lifestyle intervention remains the preferred initial recommendation for subjects with pre-diabetes and was highly effective in the DPP, not all older adults can achieve the goals of lifestyle intervention due to chronic disease, pain, and/or disability. Therefore, many older adults with pre-diabetes remain at risk for diabetes despite lifestyle intervention; in such people, pharmacotherapy for diabetes prevention may be appropriate. However, it remains unknown whether older adults with pre-diabetes are appropriate candidates for preventative pharmacotherapies—and among those therapies, which are most effective in preventing diabetes in older adults.

Current clinical guidelines from the American Diabetes Association and the American Geriatrics Society do not recommend pharmacologic agents for diabetes prevention in prediabetic adults over 60 years old (American Diabetes Association 2014). This recommendation results, in part, from a subanalysis of the DPP study which showed that, although older and younger adults responded to metformin, intensive lifestyle modification was significantly more effective than metformin in improving glucose tolerance with advancing age, as assessed by OGTT (Diabetes Prevention Program Research Group 2006). However, when conversion of pre-diabetes to diabetes was determined by an HbA1c ≥ 6.5 %, metformin was equally effective in older and younger subjects (Diabetes Prevention Program Research Group 2015). These data (Diabetes Prevention Program Research Group 2002; Sloan et al. 2008), along with the results presented here, indicate that both pioglitazone and metformin offer an alternative to delay/prevent diabetes in older subjects at high risk of developing diabetes.

It remains to be determined whether metformin might be preferable as a preventive therapy in older adults because of its other potentially beneficial anti-aging properties. Clinical trials are needed to address this question. However, evidence from animal studies suggests that metformin has aging-modulating properties such as lifespan extension (Martin-Montalvo et al. 2013), improved exercise tolerance and healthspan (Martin-Montalvo et al. 2013), and reduced incidence of cancer (Berstein 2012; DeCensi et al. 2010).

The incidence of adverse events was similar in both age groups for all outcomes. In ACT NOW, pioglitazone was associated with a small decrease in bone mineral density in the pelvis in men and women and decreased bone mineral density in the thoracic spine and ribs of women and in the lumbar spine and legs of men. However, the rate of fractures was similar in pioglitazone versus placebo groups (Bray et al. 2013; DeFronzo et al. 2011). Another study indicated that thiaozolidinediones increase fracture risk in postmenopausal women but not men (Zhu et al. 2014). Until there is more clarity about the interplay between aging, bone loss, and thiaozolidinediones, these agents should be used with caution in older subjects, particularly women.

Many issues need to be resolved before advocating routine use of thiaozolidinediones for diabetes prevention in older subjects. First, there is no evidence that pharmacologic treatment of pre-diabetes/impaired glucose tolerance or mild diabetes in older subjects improves overall morbidity and mortality. Second, the potential toxicity and adverse effects of thiaozolidinediones in older adults remain unclear. In addition to reduced bone density and possible increases in fracture risk, edema and heart failure are well-known adverse effects of thiaozolidinediones. However, in ACT NOW, overall rates of these adverse effects were low and not affected by age. Moreover, older subjects typically take many drugs, and adding another medication could increase the risk of harmful interactions. Thus, risks/benefits should be carefully assessed in every patient, particularly older adults, before prescribing drugs to prevent and treat mild diabetes (American Geriatrics Society Expert Panel on the Care of Older Adults with Diabetes Mellitus 2013). For example, an older adult who takes few medications and has good functionality and life expectancy, but shows signs of early microvascular damage (i.e., retinopathy or macroalbuminuria), may potentially benefit from an intervention for diabetes prevention/early treatment. On the other hand, in an older patient with limited life expectancy who is already taking multiple drugs, risks of additional treatment with pioglitazone may outweigh any potential benefit. Most importantly, more research in this area is required in order to make substantiated recommendations.

In summary, older prediabetic adults demonstrated similar reductions in conversion to diabetes and similar or better improvements in metabolic risk factors with pioglitazone compared to younger counterparts. Pioglitazone may be a useful pharmacologic agent to prevent diabetes in older adults.

## Electronic supplementary material

Supplemental Table 1 Baseline body composition and bone density by age group and total population (DOCX 14 kb)

Supplemental Table 2 Mean change in study outcomes in pioglitazone and placebo groups by age and for total study population (DOCX 16 kb)

Supplemental Table 3 Effect of pioglitazone versus placebo on change (baseline to study end) in body composition and bone density by age group and for total study population (DOCX 15 kb)

Supplemental Table 4 Adverse events by age group, drug assignment, and the study population overall (DOCX 15 kb)
