# Supplementary material for: Pioglitazone is equally effective for diabetes prevention in older versus younger adults with impaired glucose tolerance
Source: Age (Dordr). 2016 Sep 1;38(5-6):485–93. doi: 10.1007/s11357-016-9946-6 (PMC5266219; doi:10.1007/s11357-016-9946-6)
Supplement: Supplementary file 1 — Baseline body composition and bone density by age group and total population (DOCX 14 kb) [file 11357_2016_9946_MOESM1_ESM.docx]

| **Supplemental Table 1. Baseline body composition and bone density by age group and total population** | | | | |
| --- | --- | --- | --- | --- |
|  | **Younger**  **Age <61**  N = 194 | **Older**  **Age ≥61**  N = 38 | **Total**  N = 232 | **P-value*** |
| **Whole body fat mass (kg)** | 36.4 (11.5) | 38.6 (10.7) | 36.8 (11.4) | 0.2832 |
| **Whole body fat %** | 38.8 (7.8) | 41.9 (6.2) | 39.3 (7.6) | 0.0234 |
| **Whole body lean mass** (kg) | 56.7 (11.9) | 53.0 (10.3) | 56.0 (11.7) | 0.0776 |
| **Both arms fat mass** (kg) | 4.3 (1.9) | 4.8 (1.8) | 4.4 (1.9) | 0.0992 |
| **Both arms body fat %** | 40.5 (11.7) | 46.2 (9.8) | 41.4 (11.6) | 0.0045 |
| **Both arms lean mass** (kg ) | 6.4 (2.0) | 5.6 (1.6) | 6.2 (2.0) | 0.0324 |
| **Trunk fat mass** (kg) | 19.1 (5.9) | 19.7 (5.2) | 19.2 (5.8) | 0.5685 |
| **Trunk lean mass** (kg) | 27.8 (5.5) | 26.4 (4.6) | 27.5 (5.3) | 0.1396 |
| **Total bone density** (g/cm^2^) | 1.02 (0.11) | 0.95 (0.09) | 1.01 (0.11) | 0.0008 |
| **Whole body bone density** (including head) (g/cm^2^) | 1.16 (0.11) | 1.10 (0.09) | 1.15 (0.11) | 0.0025 |
| **Both arms bone mineral density** (g/cm^2^) | 0.82 (0.11) | 0.74 (0.08) | 0.81 (0.11) | <0.0001 |
| **Pelvis bone mineral density** (g/cm^2^) | 1.29 (0.18) | 1.23 (0.17) | 1.28 (0.18) | 0.0570 |
| **Thoracic spine** (g/cm^2^) | 0.97 (0.13) | 0.94 (0.13) | 0.97 (0.13) | 0.2010 |
