# Supplementary material for: Pioglitazone is equally effective for diabetes prevention in older versus younger adults with impaired glucose tolerance
Source: Age (Dordr). 2016 Sep 1;38(5-6):485–93. doi: 10.1007/s11357-016-9946-6 (PMC5266219; doi:10.1007/s11357-016-9946-6)
Supplement: Supplementary file 2 — Mean change in study outcomes in pioglitazone and placebo groups by age and for total study population (DOCX 16 kb) [file 11357_2016_9946_MOESM2_ESM.docx]

| **Supplemental Table 2. Mean change in study outcomes in pioglitazone and placebo groups by age and for total study population** | | | | | | | | |
| --- | --- | --- | --- | --- | --- | --- | --- | --- |
|  | **Younger**  **N = 434** | | **Older**  **N = 168** | | **Total**  **N = 602** | | **P-value for response to pioglitazone by age, unadjusted** | **P-value for response to pioglitazone by age, adjusted for sex and baseline value** |
|  | Mean (SD) | P-value | Mean (SD) | P-value | Mean (SD) | P-value |  |  |
| **Disposition index** | 1.20 (0.32) | <0.01 | 0.98 (0.57) | 0.08 | 1.13 (0.28) | 0.01 | 0.79 | 0.74 |
| **Matsuda index** | 2.54 (0.46) | <0.01 | 3.07 (0.74) | <0.01 | 2.69 (0.39) | <0.01 | 0.54 | 0.58 |
| **Fasting glucose** (mg/dL) | -0.07 (0.02) | <0.01 | -0.05 (0.02) | 0.02 | -0.06 (0.01) | <0.01 | 0.48 | 0.49 |
| **Fasting insulin** (µU/mL) | -3.19 (0.85) | <0.01 | -5.23 (1.69) | <0.01 | -3.77 (0.77) | <0.01 | 0.23 | 0.50 |
| **Hemoglobin A1c** (%) | -0.19 (0.06) | <0.01 | -0.19 (0.07) | 0.01 | -0.19 (0.05) | <0.01 | 0.75 | 0.56 |
| **IL-6** (pg/mL) | -1.72 (1.06) | 0.10 | -1.78 (1.20) | 0.14 | -1.74 (0.83) | 0.03 | 0.97 | 0.87 |
| **Leptin** (pg/mL) | -3.85 (1.77) | 0.03 | -4.43 (2.75) | 0.11 | -4.03 (1.48) | <0.01 | 0.86 | 0.84 |
| **MCP-1** (pg/mL) | -5.82 (5.31) | 0.27 | 15.6 (6.57) | 0.02 | 0.46 (4.24) | 0.19 | 0.04 | 0.02 |
| **TNF-α** (pg/mL) | -0.56 (0.27) | 0.04 | -0.21 (0.28) | 0.44 | -0.45 (0.21) | 0.03 | 0.49 | 0.44 |
| **Adiponectin** (µg/mL) | 12.70 (1.43) | <0.01 | 22.94 (3.19) | <0.01 | 15.66 (1.32) | <0.01 | 0.20 | 0.04 |
| **PAI -1** (ng/mL) | -3.43 (1.03) | <0.01 | -5.59 (1.73) | <0.01 | -4.06 (0.89) | <0.01 | 0.27 | 0.50 |
| **C-reactive protein** (mg/L) | -0.64 (0.43) | 0.14 | 0.52 (0.90) | 0.56 | -0.38 (0.44) | 0.31 | 0.21 | 0.23 |
| **HDL cholesterol** (mg/dL) | 1.41 (1.25) | 0.26 | 6.68 (2.04) | <0.01 | 2.92 (1.07) | <0.01 | 0.03 | 0.15 |
| **LDL cholesterol** (mg/dL) | -0.52 (4.24) | 0.90 | -0.25 (0.91) | 0.97 | -0.39 (3.60) | 0.91 | 0.98 | 0.99 |
| **Total cholesterol** (mg/dL) | 0.33 (4.84) | 0.94 | -2.50 (7.28) | 0.73 | 0.94 (4.02) | 0.82 | 0.81 | 0.88 |
| **Triglycerides** (mg/dL) | -10.58 (6.60) | 0.11 | -15.25 (10.5) | 0.15 | -12.28 (5.61) | 0.03 | 0.71 | 0.41 |
