# Supplementary material for: Pioglitazone is equally effective for diabetes prevention in older versus younger adults with impaired glucose tolerance
Source: Age (Dordr). 2016 Sep 1;38(5-6):485–93. doi: 10.1007/s11357-016-9946-6 (PMC5266219; doi:10.1007/s11357-016-9946-6)
Supplement: Supplementary file 3 — Effect of pioglitazone versus placebo on change (baseline to study end) in body composition and bone density by age group and for total study population (DOCX 15 kb) [file 11357_2016_9946_MOESM3_ESM.docx]

| **Supplemental Table 3. Effect of pioglitazone versus placebo on change (baseline to study end) in body composition and bone density by age group and for total study population** | | | | | | | | |
| --- | --- | --- | --- | --- | --- | --- | --- | --- |
|  | **Younger**  **Age <61**  **N = 434** | | **Older**  **Age ≥61**  **N = 168** | | **Total**  **N = 602** | | **P-value for age difference** | **P-value for age difference*** |
|  | Estimate (SE) | P | Estimate (SE) | P | Estimate  (SE) | P |  |  |
| **Both arms fat mass** (kg) | 3.21 (0.57) | <0.001 | 2.36 (1.36) | 0.092 | 3.08 (0.52) | <0.001 | 0.565 | 0.522 |
| **Both arms lean mass** (kg) | 0.47 (0.34) | 0.176 | 0.83 (0.78) | 0.298 | 0.52 (0.32) | 0.103 | 0.687 | 0.683 |
| **Whole body fat mass** (kg) | 3.62 (0.63) | <0.001 | 3.10 (1.60) | 0.061 | 3.55 (0.59) | <0.001 | 0.751 | 0.711 |
| **Whole body lean mass** (kg) | 0.32 (0.39) | 0.400 | 0.86 (0.90) | 0.347 | 0.40 (0.35) | 0.26 | 0.596 | 0.682 |
| **Trunk fat mass** (kg) | 1.41 (0.38) | <0.001 | 0.46 (0.89) | 0.608 | 1.27 (0.35) | <0.001 | 0.339 | 0.325 |
| **Trunk lean mass** (kg) | 0.20 (0.25) | 0.427 | -0.10 (0.56) | 0.857 | 0.15 (0.23) | 0.506 | 0.642 | 0.646 |
| **Both arms bone mineral density** (g/cm^2^) | .0015   (.012) | 0.900 | -.0455 (.017) | 0.010 | -0.0079 (0.007) | 0.278 | 0.121 | 0.060 |
| **Pelvis bone mineral density** (g/cm^2^) | -.066 (.013) | <0.001 | -.0114  (.06) | 0.846 | -0.055 (0.014) | <0.001 | 0.166 | 0.163 |
| **Total bone mineral density** (g/cm^2^) | -.018 ( .0071) | 0.013 | -.0138  (.021) | 0.521 | -0.017 (0.0066) | 0.011 | 0.835 | 0.843 |
| **Thoracic spine bone mineral density** (g/cm^2^) | -.029 (.015) | 0.050 | .0223  (.029) | 0.450 | -0.023( 0.012) | 0.066 | 0.850 | 0.789 |

* Adjusted for sex and baseline value
