# Supplementary material for: Pioglitazone is equally effective for diabetes prevention in older versus younger adults with impaired glucose tolerance
Source: Age (Dordr). 2016 Sep 1;38(5-6):485–93. doi: 10.1007/s11357-016-9946-6 (PMC5266219; doi:10.1007/s11357-016-9946-6)
Supplement: Supplementary file 4 — Adverse events by age group, drug assignment, and the study population overall (DOCX 15 kb) [file 11357_2016_9946_MOESM4_ESM.docx]

| **Supplemental Table 4. Adverse events by age group, drug assignment, and the study population overall** | | | | | | | | |
| --- | --- | --- | --- | --- | --- | --- | --- | --- |
|  | **Younger**  Age <61 | | | **Older**  Age >=61 | | | **Total**  N = 602 | **P-value for age difference in older vs. younger overall*** |
|  | **Pioglitazone**  N = 214 | **Placebo**  N = 220 | **P-value** | **Pioglitazone**  N = 88 | **Placebo**  N = 80 | **P-value** |  |  |
|  | N (%) or  Mean (SD) | N (%) or  Mean (SD) |  | N (%) or  Mean (SD) | N (%) or  Mean (SD) |  | N (%) or  Mean (SD) |  |
| **Change in peripheral edema** |  |  | 0.452 |  |  | 0.272 |  | 0.853 |
| -2 | 3 (1.5) | 0 (0.0) |  | 0 (0) | 1 (1.3) |  | 4 (0.7) |  |
| -1 | 28 (13.7) | 32 (15.2) |  | 13 (15.3) | 15 (19.5) |  | 88 (15.3) |  |
| 0 | 144 (70.6) | 157 (74.4) |  | 57 (67.1) | 54 (70.1) |  | 412 (71.4) |  |
| 1 | 24 (11.8) | 18 (8.5) |  | 12 (14.1) | 7 (9.1) |  | 61 (10.6) |  |
| 2 | 4 (2.0) | 3 (1.4) |  | 3 (3.5) | 0 (0.0) |  | 10 (1.7) |  |
| 3 | 1 (0.5) | 1 (0.5) |  | 0 (0.0) | 0 (0.) |  | 2 (0.4) |  |
| **Hematuria** | 13 (6.7) | 26 (13.3) | 0.071 | 7 (8.8) | 3 (4.1) | 0.445 | 49 (9.0) | 0.369 |
| **Bone Fracture** | 6 (2.8) | 6 (2.7) | 0.968 | 2 (2.3) | 1 (1.3) | 0.625 | 15 (2.5) | 0.493 |
| **Other reaction** | 13 (6.4) | 16 (7.6) | 0.842 | 4 (4.7) | 1 (1.3) | 0.176 | 34 (5.9) | 0.109 |
| **Change (actual) in body mass index** (kg/m^2^) | 0.9 (5.6) | 0.2 (3.1) | 0.129 | 1.2 (2.1) | -0.4 (4.2) | 0.002 | 0.5 (4.2) | 0.702 |
| **Change in waist size** (cm) | 3.8 (7.8) | 1.8 (7.3) | 0.019 | 3.2 (7.3) | 3.0 (6.9) | 0.893 | 2.8 (7.4) | 0.631 |

*P-value for age difference between young and older subjects overall, regardless of drug assignment.
